# Real-world use of ACEI/ARB in diabetic hypertensive patients before the initial diagnosis of obstructive coronary artery disease: patient characteristics and long-term follow-up outcome

**DOI:** 10.1186/s12967-020-02314-y

**Published:** 2020-04-01

**Authors:** Yue Zhang, Xiaosong Ding, Bing Hua, Qingbo Liu, Hui Chen, Xue-Qiao Zhao, Weiping Li, Hongwei Li

**Affiliations:** 1 Department of Cardiology, Cardiovascular Center, Beijing Friendship Hospital, Capital Medical University, 95 Yongan Road, Beijing, 100050 China; 2 Clinical Atherosclerosis Research Lab, Division of Cardiology, University of Washington, Seattle, WA USA; 3 Beijing Key Laboratory of Metabolic Disorder Related Cardiovascular Disease, Beijing, China; 4 Department of Geriatrics, Cardiovascular Center, Beijing Friendship Hospital, Capital Medical University, Beijing, China

**Keywords:** Angiotensin-converting-enzyme inhibitors/angiotensin receptor blockers (ACEI/ARB), Hypertension, Diabetes, Obstructive coronary artery disease (OCAD), Major adverse cardiac and cerebral event (MACCE)

## Abstract

**Background:**

Current guidelines recommend angiotensin-converting-enzyme inhibitors (ACEI)/angiotensin receptor blockers (ARB) as a first-line therapy in diabetic hypertensive patients and for secondary prevention in patients with obstructive coronary artery disease (OCAD). However, the effects of using ACEI/ARB before the initial diagnosis of OCAD on major adverse cardiac and cerebral event (MACCE) in diabetic hypertensive patients remain unclear. This study investigated whether using ACEI/ARB before the initial diagnosis of OCAD could be associated with improved clinical outcomes in diabetic hypertensive patients.

**Methods:**

A total of 2501 patients with hypertension and diabetes, who were first diagnosed with OCAD by coronary angiography, were included in the analysis. Of the 2501 patients, 1300 did not used ACEI/ARB before the initial diagnosis of OCAD [the ACEI/ARB(-) group]; 1201 did [the ACEI/ARB(+) group]. Propensity score matching at 1:1 was performed to select 1050 patients from each group. Incidence of acute myocardial infarction (AMI), infarct size in patients with AMI, heart function, and subsequent MACCE during a median of 25.4-month follow-up were determined and compared between the 2 groups.

**Results:**

Compared with the ACEI/ARB(-) group, the ACEI/ARB(+) group had significantly lower incidence of AMI (22.5% vs. 28.4%, p < 0.05), smaller infarct size in patients with AMI (pTNI: 5.7 vs. 6.8 ng/ml, p < 0.05; pCKMB: 21.7 vs. 28.7 ng/ml, p < 0.05), better heart function (LVEF: 60.0 vs. 58.5%, p < 0.05), and lower incidences of non-fatal stroke (2.4% vs. 4.6%, p < 0.05) and composite MACCE (23.1% vs. 29.7%, p < 0.05). No prior ACEI/ARB therapy was significantly and independently associated with non-fatal stroke and composite MACCE.

**Conclusions:**

In diabetic hypertensive patients, treatment with ACEI/ARB before the initial diagnosis with OCAD was associated with decreased incidence of AMI, smaller infarct size, improved heart function, and lower incidences of non-fatal stroke and composite MACCE.

*Trial registration* Retrospectively registered

## Background

Cardiovascular disease is the leading cause of death worldwide [1–3], especially obstructive coronary artery disease (OCAD). Hypertension and diabetes are strong independent risk factors for OCAD and associated with most of the cardiovascular death globally [4, 5]. Individuals with both hypertension and diabetes are at a higher risk of OCAD than those with either of the two conditions [6].

It has been well known that the renin-angiotensin-aldosterone system (RAAS) plays an important role in regulating cardiovascular and renal function [7, 8]. Randomized clinical trials have confirmed that suppression of RAAS activity by angiotensin-converting-enzyme inhibitors/angiotensin receptor blockers (ACEI/ARB) can protect cardio-renal function and reduce mortality [9–13]. Thus, the current guidelines recommend ACEI/ARB as a first-line therapy for diabetic hypertensive patients [14–16] and for secondary prevention in patients with OCAD [17, 18].

It has been generally accepted that diabetic hypertensive patients can benefit from ACEI/ARB; however, previous studies have found the ACEI/ARB is underutilized in these patients [19–21]. The real-world use of ACEI/ARB in diabetic hypertensive patients in China remains unclear. Diabetic hypertensive patients are prone to develop OCAD. Most previous studies have emphasized the secondary preventive effects of ACEI/ARB on OCAD. Whether starting ACEI/ARB therapy before the initial diagnosis of OCAD could improve patient outcomes is still unknown. The current study aimed to fill this knowledge gap. We used the Cardiovascular Center Beijing Friendship Hospital Database Bank to evaluate the effectiveness of ACEI/ARB therapy on improving major adverse cardiac and cerebral event (MACCE) outcomes in diabetic hypertensive patients.

## Methods

### Study population

**Fig. 1:**
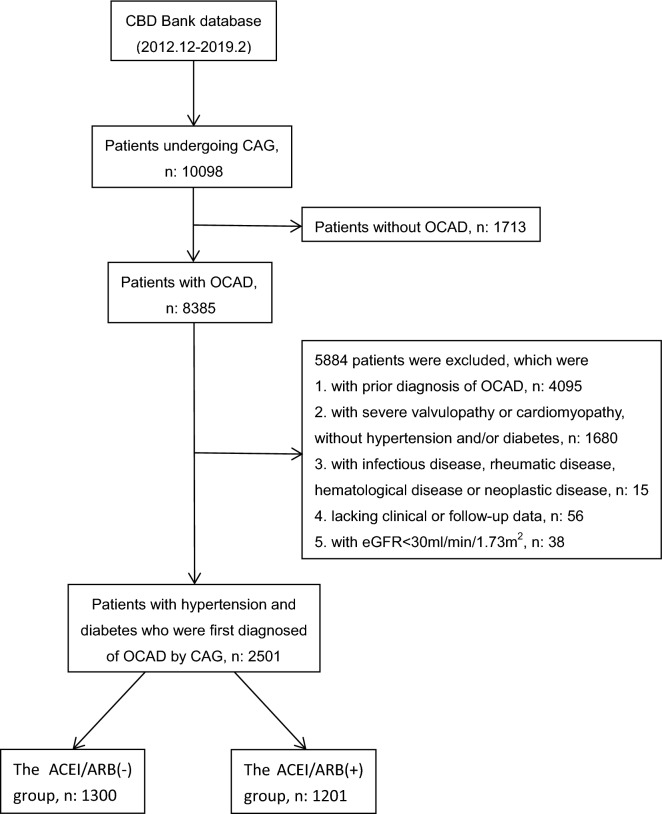
Patient flow chart. *CBD* Cardiovascular Center of Beijing Friendship Hospital Database, *CAG* coronary angiography, *OCAD* obstructive coronary artery disease, *eGFR* estimated glomerular filtration rate, *ACEI/ARB* angiotensin-converting enzyme inhibitors/angiotensin receptor blockers

Patients’ records in the Cardiovascular Center of Beijing Friendship Hospital Database Bank were screened. As shown in Fig. 1, the records of 10,098 patients undergoing coronary angiography from December 2012 to February 2019 in our center were screened. Of them, 8385 patients were diagnosed with OCAD. Of the 8385 patients, 5884 were excluded according to the exclusion criteria, which were (1) with prior diagnosis of OCAD, (2) with severe valvulopathy or cardiomyopathy and without hypertension and/or diabetes, (3) with acute infections disease, rheumatic disease, hematological disease, or neoplastic disease, (4) lacking clinical or follow-up data, and (5) with estimated glomerular filtration rate(eGFR) < 30 ml/min/1.73 m^2^. Finally, 2501 patients were included in this analysis. Of the 2501 patients, 1300 were not treated with ACEI/ARB before the initial diagnosis of OCAD; 1201 were confirmed to receive ACEI/ARB treatment before the diagnosis. All patients were followed up to May 31, 2019 with a median follow up of 25.4 months (IQR: 12.3, 48.6 months).

### Data collections and definitions

The data collection process was approved by the Institutional Review Board of Beijing Friendship Hospital affiliated to Capital Medical University and was in accordance with the Declaration of Helsinki.

Patients’ demographics, medical and medication history, laboratory test results, echocardiographic and angiographic evaluation results, and clinical outcomes during the hospitalization after the initial diagnosis of OCAD were collected and verified using an electronic medical recording system. The outcomes from MACCE were collected and recorded during clinical follow-up visits.

MACCE included all-cause death, non-fatal myocardial infarction (MI), non-fatal stroke, revascularization, and cardiac rehospitalization (admission because of angina or heart failure). All-cause death was defined as the incidence of cardiovascular death or non-cardiovascular death. Cardiovascular death was defined as fatal stroke and MI, sudden death, and other cardiovascular death. Any coronary revascularization was defined as a revascularization of the target vessel or non-target vessels. Non-fatal MI was defined as chest pain with new ST-segment changes and elevation of myocardial necrosis markers to at least twice of the upper limit of the normal range. Non-fatal stroke, including ischemic and hemorrhagic stroke, was defined as cerebral dysfunction caused by cerebral vascular obstruction or sudden rupture and was diagnosed based on signs of neurological dysfunction or evidence of brain imaging. Cardiac rehospitalization refers to rehospitalization for angina pectoris or heart failure.

### Statistical analyses

Continuous variables are presented as mean ± standard deviation (SD) or median (IQR). Comparisons between the two study groups were analyzed by Student’s *t* test or Mann–Whitney U-test. Categorical variables are expressed as number and percentage and compared using the Pearson Chi square test or Fisher’s exact test. To control confounding factors, we performed propensity score matching. The cumulative incidence of MACCE was estimated by Kaplan–Meier survival curves. A multivariable Cox regression analysis was performed to identify independent predictors for composite MACCE. Baseline variables that were significantly correlated with outcomes by univariate analysis and clinically relevant were used in the multivariate model. For the COX regression, the outcome event is at least 15–20 times the number of variables. Thus, the included variables for the COX regression were carefully chosen, given the number of events available, to ensure parsimony of the final model. All analyses were two-tailed and P value < 0.05 was considered statistically significant.

### Propensity score matching

Propensity score matching was used to reduce selection bias in this study. The matching process was conducted with a minimum-distance scoring method and a 1-to-1 match between the ACEI/ARB(-) group and the ACEI/ARB(+) group. In this study, propensity scores were calculated through a binary logistic regression model, including covariates of age, sex, body mass index (BMI), fasting blood glucose (FBG), hemoglobin (HGB), eGFR, low-density lipoprotein cholesterol (LDL-C), history of smoking and dyslipidemia, previous medication history including antiplatelet agent, beta-blocker, and statins. Ultimately, 1050 ACEI/ARB(+) patients were individually 1:1 matched to 1050 ACEI/ARB(-) controls using nearest available score matching. The statistical analysis software SPSS version 24.0 was used for the matching.

## Results

### Patient characteristics

As shown in Fig. 1, of the 2501 eligible patients, 1201 patients (48.0%) used ACEI/ARB before the hospital admission; 1300 (52.0%) did not. Comparing with the ACEI/ARB (+) group, the ACEI/ARB (-) group showed significantly higher percent of male, lower BMI, higher heart rate, lower percent of dyslipidemia, and significantly less likely to receive antiplatelet therapy, beta-blocker or statins before the hospital admission for OCAD. In-hospital medical and interventional treatments were similar between the 2 groups except that significantly fewer patients treated with ACEI/ARB in the ACEI/ARB (-) group than in the ACEI/ARB (+) group (52.1% vs. 83.9%, p < 0.001) during hospitalization. Subjects in the ACEI/ARB (-) group had a significant longer average hospital stay (Table 1).

**Table 1:** Baseline clinical characteristics

Characteristics	Before PS match		P value	After PS match		P value
	ACEI/ARB(-)	ACEI/ARB(+)		ACEI/ARB(-)	ACEI/ARB(+)	
	(n: 1300)	(n: 1201)		(n: 1050)	(n: 1050)	
Age, years	64.7 ± 10.5	65.2 ± 10.0	NS	65.8 ± 11.5	65.4 ± 12.2	NS
Male	774 (59.5)	662 (55.1)	< 0.05	578 (55.0)	592 (56.4)	NS
BMI, kg/m^2^	26.2 ± 3.5	26.6 ± 3.5	<0.001	25.9 ± 3.4	26.2 ± 3.5	NS
SBP, mmHg	136.0 ± 19.7	135.0 ± 18.9	NS	134.9 ± 22.4	134.3 ± 21.1	NS
DBP, mmHg	76.9 ± 12.3	75.7 ± 11.5	NS	74.8 ± 12.3	74.5 ± 13.3	NS
Heart rate, bpm	74.1 ± 13.0	71.9 ± 12.1	<0.001	74.7 ± 13.2	74.7 ± 12.8	NS
Medical history						
Current/ex-smoker	628 (48.3)	535 (44.5)	NS	473 (45.0)	479 (45.6)	NS
CKD	34 (2.6)	43 (3.6)	NS	29 (2.8)	36 (3.4)	NS
Stroke	239 (18.4)	243 (20.2)	NS	203 (19.3)	207 (19.7)	NS
Dyslipidemia	580 (44.6)	603(50.2)	<0.05	478 (45.5)	492 (46.9)	NS
Medication used before admission						
Antiplatelet agent	336 (25.8)	428 (35.6)	< 0.001	312 (29.7)	332 (31.6)	NS
Beta-blocker	283 (21.8)	356 (30.6)	< 0.001	265 (25.2)	280 (26.7)	NS
CCB	703 (54.1)	617 (51.4)	NS	583 (55.5)	543 (51.7)	NS
Diuretics	42 (3.2)	51 (4.2)	NS	34 (3.2)	45 (4.3)	NS
Statins	319 (24.5)	457 (38.1)	< 0.001	315 (30.0)	330 (31.4)	NS
In-hospital treatment						
PCI/CABG	918 (70.6)	818 (68.1)	NS	719 (68.5)	723 (68.9)	NS
Antiplatelet agent	1249 (96.1)	1152 (95.9)	NS	1007 (95.9)	1005 (95.7)	NS
ACEI/ARB	677 (52.1)	1008 (83.9)	<0.001	535 (51.0)	881 (83.9)	< 0.001
Beta-blocker	940 (72.3)	849 (70.7)	NS	760 (72.4)	742 (70.7)	NS
CCB	586 (45.1)	574 (47.8)	NS	485 (46.2)	502 (47.8)	NS
Diuretics	94 (7.1)	81(6.7)	NS	79 (7.5)	75 (7.1)	NS
Statins	1151 (88.5)	1075 (89.5)	NS	935 (89.0)	937 (89.2)	NS
Hospital stay, day	6 (5.8)	6 (4.8)	< 0.05	6 (4.8)	6 (4.8)	NS

Data are presented as mean ± SD, IQR or n (%)

*ACEI/ARB* angiotensin-converting enzyme inhibitor/angiotensin receptor blocker, *BMI* body mass index, *SBP* systolic blood pressure, *DBP* diastolic blood pressure, *CKD* Chronic kidney disease, *CCB* calcium channel blocker, *PCI* percutaneous coronary intervention, *CABG* coronary artery bypass graft, *NS* non-significant

As presented in Table 2, the ACEI/ARB(-) group had significantly higher white cell count, neutrophil count and higher levels of sensitivity C reactive protein (hsCRP), HGB, FBG, random blood glucose(RBG) at admission, eGFR, and LDL-C than the ACEI/ARB(+) group. Echo evaluation showed that the ACEI/ARB(-) group had significantly greater left ventricular end-systolic diameter (LVESD) and left ventricular end-systolic volume (LVESV) and lower left ventricular ejection fraction (LVEF), left ventricular fraction shortening (LVFS) and stroke volume (SV) than the ACEI/ARB(+) group. Angiographically, there was no significant difference between the 2 groups.

**Table 2:** Laboratory test results and echocardiographic and angiographic characteristics

	Before PS match		P value	After PS match		P value
	ACEI/ARB(-)	ACEI/ARB(+)		ACEI/ARB(-)	ACEI/ARB(+)	
	(n: 1300)	(n: 1201)		(n: 1050)	(n: 1050)	
Laboratory values						
WBC, X10^9^/L	6.8 (5.6, 8.7)	6.6 (5.5, 8.1)	<0.05	8.4 (6.3, 10.1)	7.9 (6.1, 10.2)	NS
Neutrophil, X10^9^/L	4.5 (3.6, 5.9)	4.4 (3.5, 5.5)	<0.05	5.9 (4.3, 7.7)	5.5 (4.0, 7.7)	NS
Monocyte, X10^9^/L	0.29 (0.16, 0.44)	0.27 (0.16, 0.41)	NS	0.27 (0.15, 0.42)	0.28 (0.16, 0.41)	NS
Hemoglobin, g/L	134.5 ± 16.5	133.7 ± 15.7	<0.05	133.0 ± 18.9	132.9 ± 19.1	NS
Hs-CRP, mg/L	2.7 (1.0, 10.4)	2.0 (0.8, 5.4)	<0.001	10.5 (2.9, 23.3)	6.1 (2.0, 16.7)	<0.05
FBG, mmol/L	7.0 (5.8, 9.0)	6.8 (5.7, 8.3)	<0.05	7.5 (6.2, 9.3)	7.5 (6.3, 10.2)	NS
RBG at admission, mmol/L	9.9 (7.5, 13.3)	9.7 (7.2, 12.8)	<0.05	10.8 (8.1, 14.6)	11.0 (8.7, 14.2)	NS
Glycated hemoglobin, %	7.5 ± 1.5	7.5 ± 1.6	NS	7.7 ± 1.7	7.8 ± 1.7	NS
ALT, U/L	19.0 (13.0, 28.0)	18.0 (13.0, 27.0)	NS	22.0 (15.0, 39.0)	21.0 (14.0, 35.0)	NS
Creatinine, µmol/L	76.0 (65.3, 88.1)	77.8 (66.2, 90.6)	<0.05	81.8 (70.6, 96.9)	81.0 (68.7, 94.0)	NS
eGFR, ml/min/1.73 m^2^	84.9 (71.4, 97.9)	82.3 (67.9, 95.3)	<0.001	79.1 (64.5, 94.5)	79.2 (64.0, 95.2)	NS
TC, mmol/L	4.3 (3.6, 5.0)	4.2 (3.4, 4.9)	NS	4.45 ± 0.97	4.48 ± 1.12	NS
TG, mmol/L	1.5 (1.1, 2.2)	1.5 (1.1, 2.1)	NS	1.5 (1.1, 2.0)	1.7 (1.2, 2.3)	NS
LDL-C, mmol/L	2.4 (1.9, 2.9)	2.3 (1.8, 2.8)	< 0.001	2.6 (2.1, 3.1)	2.6 (2.0, 3.1)	NS
HDL-C, mmol/L	1.0 (0.9, 1.2)	1.0 (0.9, 1.2)	NS	1.0 (0.9, 1.2)	1.0 (0.8, 1.2)	NS
NT-Pro BNP,pg/ml	1621(644, 4214)	1585(521, 4548)	NS	1855(657, 4571)	1585(487, 4357)	NS
Echocardiographic values						
LVEDD, cm	5.06 ± 0.49	5.04 ± 0.48	NS	5.13 ± 0.51	5.17 ± 0.52	NS
LVESD, cm	3.28 ± 0.57	3.21 ± 0.51	<0.05	3.53 ± 0.59	3.51 ± 0.61	<0.05
LVEF, %	63.93 ± 8.92	65.66 ± 7.51	<0.001	58.5 ± 9.4	60.0 ± 9.7	<0.05
LVFS, %	35.33 ± 6.22	36.52 ± 5.92	<0.001	31.4 ± 6.2	32.8 ± 7.2	<0.05
LVEDV, ml	122.9 ± 28.3	122.2 ± 27.9	NS	127.5 ± 30.0	129.9 ± 31.9	NS
LVESV, ml	41.0 (32.2, 51.2)	38.2 (32.2, 48.4)	<0.05	47.4 (38.8, 65.9)	47.4 (36.7, 62.0)	NS
LA,cm	3.71 ± 0.43	3.74 ± 0.41	<0.05	3.76 ± 0.45	3.78 ± 0.42	NS
E/A	0.80 (0.68, 1.00)	0.79 (0.68, 1.00)	NS	0.84 (0.69, 1.16)	0.81 (0.67, 1.16)	NS
SV, ml	76.7 (66.5, 87.9)	79.2 (68.7, 88.9)	<0.05	70.8 (63.4, 83.7)	75.4 (65.4, 87.1)	NS
Angiography values						
Involved vessel						
Single vessel	180 (13.8)	158 (13.2)	NS	157 (15.0)	138 (13.1)	NS
Multi-vessel/LM	1120 (86.2)	1043 (86.8)		893 (85.0)	912 (86.9)	
CTO	122 (9.4)	108 (9.0)	NS	92 (8.8)	88 (8.4)	NS
Proximal LAD	697 (53.6)	626 (52.1)	NS	557 (53.0)	551 (52.5)	NS

Data are presented as mean ± SD, IQR or n (%)

*WBC* white blood cell count, *Hs-CRP* high sensitivity C reactive protein, *FBG* fasting blood glucose, *RBG* random blood glucose, *ALT* alanine transaminase, *eGFR* estimated glomerular filtration rate, *TC* total cholesterol, *TG* triglycerides; *LDL-C* low-density lipoprotein cholesterol, *HDL-C* high-density lipoprotein cholesterol, *NT-Pro BNP* N-terminal pro-brain natriuretic peptide, *LVEDD* left ventricular end-diastolic diameter, *LVESD* left ventricular end-systolic diameter, *LVEF* left ventricular ejection fraction, *LVFS* left ventricular fraction shortening, *LVEDV* left ventricular end-diastolic volume, *LVESV* left ventricular end-systolic volume, *LA* left atrium, *E/A* ratio of early to late ventricular filling velocities, *SV* stroke volume, *LM* left main coronary artery, *CTO* chronic total occlusions, *LAD* left anterior descending, *NS* non-significant

**Fig. 2:**
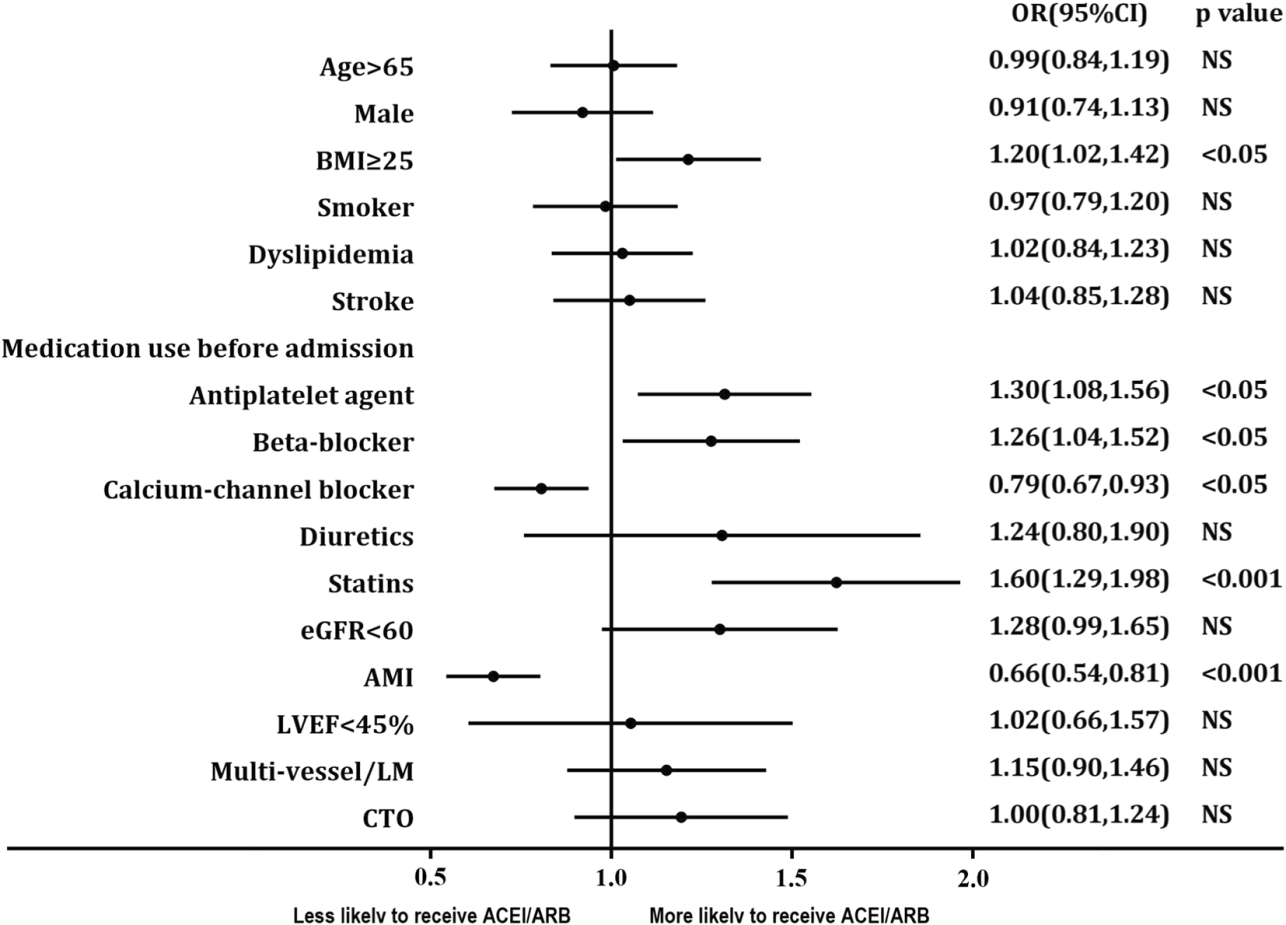
Factors associated with ACEI/ARB use in multivariable analysis. Variables associated with ACEI/ARB use are shown along the vertical axis. The strength of effect is shown along the horizontal axis with the vertical line demarcating an odds ratio (OR) of 1 (i.e., no association); estimates to the right (i.e., > 1) are associated with a greater likelihood of ACEI/ARB use, whereas those to the left (i.e., < 1) indicate a reduced likelihood of ACEI/ARB use. Each dot represents the point estimate of the effect of that variable in the model, whereas the line shows the 95% confidence interval (CI). *BMI* body mass index, *eGFR* estimated Glomerular filtration rate, *AMI* acute myocardial infarction, *LVEF* left ventricular ejection fraction, *LM* left main coronary artery, CTO chronic total occlusions, *NS* Non-significant

Correlation analysis of ACEI/ARB therapy and baseline variables revealed that patients with BMI ≥ 25 kg/m^2^, previous use of antiplatelet agent, beta-blocker and statins were more likely to receive ACEI/ARB therapy before the hospital admission. However, patients with AMI at admission and a previous history of using calcium channel blocker (CCB) were less likely to receive ACEI/ARB therapy before the hospital admission (Fig. 2).

### Propensity score matching

Propensity scores of 1050 ACEI/ARB users were 1:1 matched to 1050 patients without using ACEI/ARB before the initial diagnosis of OCAD. There were no significant differences in baseline clinical characteristics and medical history between the propensity score matched (PSM) ACEI/ARB(-) and ACEI/ARB(+) groups except that the PSM ACEI/ARB(-) group had significantly fewer patients treated with ACEI/ARB therapy during the hospitalization (51.0% vs. 83.9%, p < 0.001, Table 1).

The ACEI/ARB(-) group had significantly higher hsCRP levels than the ACEI/ARB(+) group. Echo evaluation showed that the ACEI/ARB(-) group had significantly larger LVESD, lower LVEF and LVFS than the ACEI/ARB(+) group (Table 2).

**Fig. 3:**
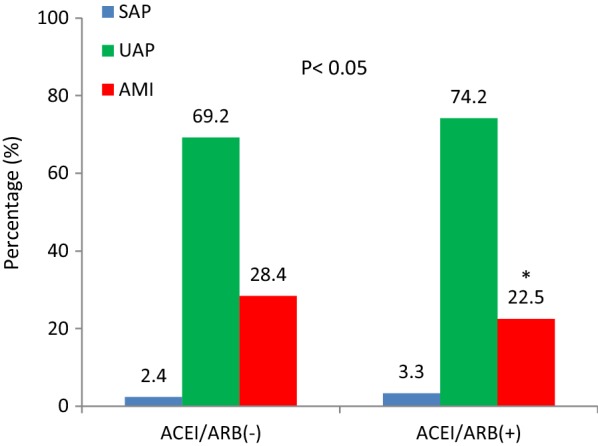
Percentages of patients with SAP, UAP and AMI in 2 groups. *SAP* stable angina pectoris, *UAP* unstable angina pectoris, *AMI* acute myocardial infarction, *ACEI/ARB* angiotensin-converting enzyme inhibitor/angiotensin receptor blocker. *P < 0.05 versus ACEI/ARB(-) group

The ACEI/ARB(-) group had a significantly higher incidence of acute myocardial infarction(AMI) at the admission than the ACEI/ARB(+) group (28.4% vs. 22.5%, p < 0.05, Fig. 3). The peak levels of serum myoglobin (Myo), creatine kinase MB (CKMB), and cardiac troponin I (cTnI) were used to estimate infarct size. We found no difference in pMyo between the 2 groups. The peak levels of serum CKMB and cTnI were significantly higher in the ACEI/ARB(-) group (p-CKMB: 28.7 vs. 21.7 ng/mL, p < 0.05; p-cTnI: 6.8 vs. 5.7 ng/mL, p < 0.05, Table 3).

**Table 3:** The estimated infarction size in patients with AMI

The peak value of myocardial enzyme	Before PS match		P value	After PS match		P value
	ACEI/ARB(-)	ACEI/ARB(+)		ACEI/ARB(-)	ACEI/ARB(+)	
	(n: 402)	(n: 251)		(n: 298)	(n: 236)	
pMYO,ng/ml	50.1 (26.0, 150.3)	46.0 (17.4, 146.5)	NS	50.4 (28.3, 173.8)	46.1 (17.8, 150.0)	NS
pCK-MB,ng/ml	28.4 (8.0, 116.0)	21.3 (5.2, 89.2)	< 0.05	28.7 (8.2, 119.3)	21.7 (5.2, 90.9)	< 0.05
pTNI,ng/ml	7.7 (2.3, 27.0)	5.4 (1.0, 22.5)	< 0.05	6.8 (2.2, 22.9)	5.7 (1.0, 24.3)	< 0.05

Data are presented as IQR

*AMI* acute myocardial infarction, *ACEI/ARB* angiotensin-converting enzyme inhibitor/angiotensin receptor blocker, *pMYO* the peak value of myoglobin, *pCK-MB* the peak value of creatine kinase MB, *pTNI* the peak value of troponin I, *NS* non-significant

### In-hospital clinical outcomes

The ACEI/ARB(-) group had significantly higher incidence of non-fatal stroke than the ACEI/ARB(+) group (Before propensity score matching: 1.6% vs. 0.7%, p < 0.05; After propensity score matching:1.7% vs. 0.8%, p < 0.05). There was no statistical difference in the other MACCE between the 2 groups.

### Subsequent MACCE and mortality

During a median of 25.4 months (IQR: 12.3, 48.6 months) follow-up, composite MACCE occurred in 28.7% of patients in the ACEI/ARB (-) group and 23.1% in the ACEI/ARB (+) group (HR = 1.23, 95%CI 1.06, 1.44, p < 0.05, Table 4). Non-fatal stroke occurred in 4.0% of the patients in the ACEI/ARB (-) group and 2.4% in the ACEI/ARB (+) group (HR = 1.62, 95%CI 1.03, 2.56, p < 0.05). The incidences of all cause death, cardiovascular death, non-fatal MI, revascularization, and cardiac rehospitalization were not statistically different between the 2 groups.

**Table 4:** Clinical events during long-term follow-up

	ACEI/ARB(-)	ACEI/ARB(+)	HR(95%CI)	P value
Overall population				
Number	1300	1201		
Composite MACCE	373 (28.7)	277 (23.1)	1.23 (1.06,1.44)	<0.05
All cause death	59 (4.5)	57 (4.7)	0.92 (0.64,1.32)	NS
CV death	49 (3.8)	37 (3.1)	1.18 (0.77,1.81)	NS
Non-fatal MI	57 (4.4)	37 (3.1)	1.38 (0.91,2.08)	NS
Non-fatal stroke	52 (4.0)	29 (2.4)	1.62 (1.03,2.56)	<0.05
Revascularization	102 (7.8)	92 (7.7)	0.98 (0.74,1.29)	NS
Cardiac rehospitalization	290 (22.3)	233 (19.4)	1.12 (0.94, 1.33)	NS
Matched population				
Number	1050	1050		
Composite MACCE	312 (29.7)	242 (23.1)	1.21 (1.02, 1.43)	<0.05
All cause death	53 (5.0)	51 (4.9)	0.95 (0.65, 1.40)	NS
CV death	44 (4.2)	33 (3.1)	1.24 (0.79, 1.94)	NS
Non-fatal MI	48 (4.6)	31 (3.0)	1.45 (0.92, 2.28)	NS
Non-fatal stroke	48 (4.6)	25 (2.4)	1.82 (1.13, 2.96)	<0.05
Revascularization	76 (7.2)	81 (7.7)	0.85 (0.62, 1.16)	NS
Cardiac rehospitalization	241 (23.0)	202 (19.2)	1.09 (0.91, 1.32)	NS

*ACEI/ARB* angiotensin-converting enzyme inhibitor/angiotensin receptor blocker, *MACCE* major adverse cardiac and cerebral event, *CV* cardiovascular, *MI m*yocardial infarction, *HR* hazard ratio, *CI* confidence interval, *NS* non-significant

**Fig. 4:**
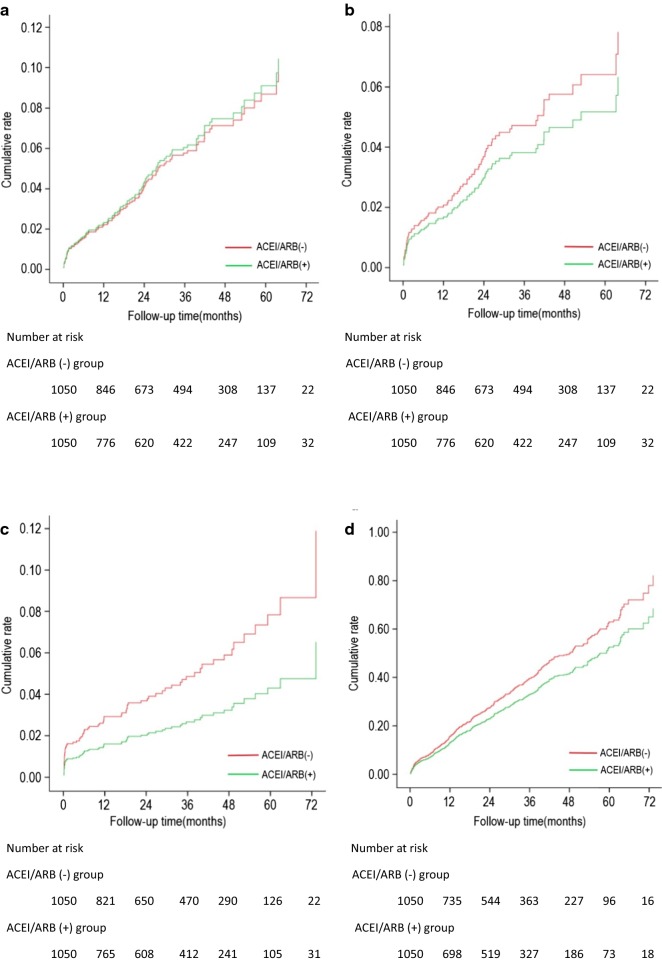
Kaplan-Meier curve for all cause death (**a**) CV death (**b**) non-fatal stroke (**c**) and composite MACCE (**d**) of the ACEI/ARB(-) group (red line) versus the ACEI/ARB(+) group (green line). **a** There was no significant difference in the cumulative rate of all cause death between the 2 groups. **b** There was no significant difference in the cumulative rate of CV death between the 2 groups. **c** The cumulative rate of non-fatal stroke in the ACEI/ARB(-) group was significantly higher than that in the ACEI/ARB(+) group(p < 0.05). **d **The cumulative rate of composite MACCE in the ACEI/ARB(-) group was significantly higher than that in the ACEI/ARB(+) group (p < 0.05). *ACEI/ARB* angiotensin-converting enzyme inhibitor/Angiotensin receptor blocker, *CV* Cardiovascular, *MACCE* major adverse cardiac and cerebral event

After propensity-score matching, composite MACCE occurred in 29.7% of the patients in the PSM ACEI/ARB(-) group and 23.1% in the PSM ACEI/ARB(+) group (HR = 1.21, 95%CI 1.02, 1.43, p < 0.05, Table 4); non-fatal stroke occurred in 4.6% of the PSM ACEI/ARB (-) group and 2.4% of the PSM ACEI/ARB (+) group (HR = 1.82, 95%CI 1.13, 2.96, p < 0.05). The incidences of all cause death, cardiovascular death, non-fatal MI, revascularization, and cardiac rehospitalization were not statistically different between the 2 groups. The Kaplan–Meier curves show that the ACEI/ARB(-) group had significantly higher cumulative rate of non-fatal stroke and composite MACCE than the ACEI/ARB (+) group (Fig. 4).

### Independent association between non-fatal stroke and subsequent MACCE

In the multivariate analysis, we included variables that were identified to be significantly associated with non-fatal stroke and composite MACCE in the univariate model. The multivariate analysis revealed that no prior ACEI/ARB therapy, previous history of stroke, increased number of involved vessels, and lower LVEF were independently associated with non-fatal stroke (Table 5); no prior ACEI/ARB therapy, previous history of stroke, increased number of involved vessels, lower eGFR, lower LVEF, and no-antiplatelet therapy in hospital were significantly and independently associated with subsequent composite MACCE (Table 6).

**Table 5:** Multivariate COX regression analysis of non-fatal stroke

	Univariate		Multivariate	
	HR (95%CI)	P value	Adjusted HR (95%CI)	P value
Age, y	1.02 (0.99, 1.05)	NS	0.99 (0.97, 1.02)	NS
ACEI/ARB(-)	1.82 (1.13, 2.96)	< 0.05	1.72 (1.05, 2.84)	< 0.05
Beta-blocker before admission	0.54 (0.29, 1.01)	NS	0.56 (0.30, 1.06)	NS
Previous stroke	4.00 (2.52, 6.35)	< 0.001	3.70 (2.29, 5.96)	< 0.001
Hemoglobin, g/L	0.98 (0.96, 0.99)	< 0.05	0.99 (0.97, 1.01)	NS
AMI at admission	2.10 (1.32, 3.35)	< 0.05	1.07 (0.61, 1.86)	NS
LVEF, %	0.95 (0.93, 0.97)	< 0.001	0.96 (0.94, 0.99)	< 0.05
Involved vessel	1.79 (1.28, 2.49)	< 0.05	1.52 (1.08, 2.14)	< 0.05
In-hospital treatment				
ACEI/ARB	0.72 (0.46, 1.14)	NS	0.85 (0.52, 1.38)	NS
Antiplatelet agents	0.34 (0.16, 0.75)	<0.05	0.49 (0.19, 1.25)	NS
Statins	0.43 (0.24, 0.76)	<0.05	0.58 (0.30, 1.11)	NS

*ACEI/ARB(-)* no Angiotensin-converting enzyme inhibitor/Angiotensin receptor blocker therapy before admission, *AMI* acute myocardial infarction, *LVEF* left ventricular ejection fraction; *NS* Non-significant

**Table 6:** Multivariate COX regression analysis of composite MACCE

	Univariate		Multivariate	
	HR(95%CI)	P value	Adjusted HR(95%CI)	P value
Age,y	1.01 (0.99, 1.02)	NS	1.00 (0.99, 1.01)	NS
Male, %	1.05 (0.89, 1.25)	NS	1.05 (0.82, 1.33)	NS
ACEI/ARB(-)	1.21 (1.02, 1.43)	< 0.05	1.24 (1.04, 1.48)	< 0.05
Previous stroke	1.57 (1.29, 1.90)	< 0.001	1.49 (1.22, 1.82)	< 0.001
Smoking	1.09 (0.93, 1.29)	NS	1.05 (0.85, 1.31)	NS
Hemoglobin, g/L	1.00 (0.99, 1.00)	NS	0.99 (0.98, 1.00)	NS
Glycated hemoglobin, %	1.08 (1.02, 1.14)	< 0.05	1.05 (0.99, 1.11)	NS
LDL-C, mmol/L	1.15 (1.03, 1.28)	< 0.05	1.07 (0.95, 1.20)	NS
eGFR ml/min/1.73 m^2^	0.98 (0.97, 0.99)	< 0.001	0.98 (0.97, 0.99)	< 0.05
AMI at admission	1.62 (1.36, 1.93)	< 0.001	1.18 (0.96, 1.45)	NS
LVEF, %	0.97 (0.96, 0.98)	< 0.001	0.98 (0.97, 0.99)	< 0.001
Involved vessel	1.45 (1.29, 1.62)	< 0.001	1.34 (1.19, 1.51)	<0.001
CTO	1.49 (1.23, 1.81)	< 0.001	1.07 (0.87, 1.33)	NS
In-hospital treatment				
Antiplatelet agents	0.45 (0.33, 0.63)	< 0.001	0.45 (0.31, 0.65)	< 0.001
ACEI/ARB	0.92 (0.78, 1.09)	NS	1.00 (0.84, 1.19)	NS
Beta-blocker	0.93 (0.78, 1.12)	NS	0.87 (0.72, 1.06)	NS
Statins	0.75 (0.58, 0.95)	<0.05	0.94 (0.72, 1.23)	NS

*ACEI/ARB(-)* no angiotensin-converting enzyme inhibitor/angiotensin receptor blocker therapy before admission, *LDL-C l*ow-density lipoprotein cholesterol, *eGFR* estimated glomerular filtration rate, *AMI* acute myocardial infarction, *LVEF* left ventricular ejection fraction, *CTO* chronic total occlusions; *NS* non-significant

## Discussion

To the best of our knowledge, the current study was the first to investigate whether ACEI/ARB used before the initial diagnosis of OCAD in diabetic hypertensive patients could be associated with improved clinical outcomes. We found that use of ACEI/ARB before the initial diagnosis of OCAD was associated with reduced incidence of AMI, reduced myocardial infarction size, and improved cardiac function, whereas we found no significant correlation between the prior ACEI/ARB therapy and mortality. However, the incidences of non-fatal stroke and composite MACCE were significantly higher in the ACEI/ARB(-) group than in the ACEI/ARB(+) group. No prior ACEI/ARB therapy was an independent predictor of non-fatal stroke and composite MACCE.

ACEI promotes vasodilation by inhibiting angiotensin II formation and bradykinin decomposition, and ARB can trigger vasodilation and natriuresis. Therefore, ACEI/ARB are considered as antihypertensive drugs. In addition to the antihypertensive effects, ACEI/ARB has other pleiotropic clinical beneficial effects, such as inhibiting ventricular remodeling, decreasing sympathetic activity, improving insulin resistance, inhibiting atherosclerosis process, inhibiting thrombosis and platelet aggregation, and improving endothelium function and plaque stabilization [22–25]. Previous studies [11, 12, 26] have supported that ACEI/ARB exert clinical beneficial effects beyond blood pressure reduction and can reduce the incidence of major adverse cardiac events.

The current guidelines recommend ACEI/ARB as a first-line drug for diabetic hypertensive patients [14–16]; however, the reported effects of ACEI/ARB on cardiovascular risk in these patients are controversial. Previous studies have found that patients treated with ACEI/ARB showed lower incidences of AMI [27–29] and stroke [28, 30, 31] than the control group. In addition, the Captopril Prevention Project (CAPPP) [29] has shown that compared with the diuretic/beta-blocker therapy group, the captopril group had lower incidences of cardiovascular mortality and all-cause mortality. A meta-analysis has demonstrated that ACEI/ARB was associated with a 17% reduction in cardiovascular mortality in diabetic hypertensive patients; however, ACEI/ARB was not associated with MI, stroke and all-cause mortality [32]. On the contrary, Bosch et al. [33] have shown that ACEI was not beneficial in the prevention of stroke. In addition, the Candesartan Antihypertensive Survival Evaluation in Japan (CASE-J) trial, which recruited 2018 patients with T_2_DM, failed to find a reduction in cardiovascular morbidity in patients using ARB [34]. A recent meta-analysis [12] has shown that ACEI significantly reduced the risk of AMI, cardiovascular mortality, and all-cause mortality, whereas treatment with ARBs did not show these benefits. In addition, neither ACEI nor ARB therapy decreased the incidence of stroke in patients with diabetes. Strauss et al. [35] believed that ARB could not reduce the risk of AMI, cardiovascular mortality, or all-cause Mortality. In the current study, we found that compared with diabetic hypertensive patients who did not use ACEI/ARB before the initial diagnosis of OCAD, those who did had significantly lower incidence of OCAD-associated AMI at the hospital admission and lower incidences of non-fatal stroke and composite MACCE during follow-up. Multivariate analyses revealed that no prior ACEI/ARB therapy was an independent predictor of non-fatal stroke and composite MACCE. These findings have not been reported previously. There were 249 patients (23.7%) using ACEI and 801 patients (76.3%) using ARB before the initial diagnosis of OCAD in the PSM ACEI/ARB(+) group in this study. The incidence of AMI in the ACEI users and ARB users was 24.1% (60/249) and 22.0% (176/801), respectively, and the incidence of AMI in the PSM ACEI/ARB(-) group was 28.4% (298/1050). Therefore, we believed that both ACEI and ARB can reduce AMI development.

Notably, we found that the ACEI/ARB(-) group had significantly higher levels of hs-CRP, pTNI, and pCK-MB and lower LVEF than the ACEI/ARB(+) group, indicating that the patients who did not use ACEI/ARB before the initial diagnosis of OCAD appeared to have higher levels of inflammation, larger myocardial infarction area, and poorer cardiac function. Consistently, Gong et al. [36] also found that previous treatment with ACEI/ARB/β-blocker was associated with better heart function and smaller infarct size. To the best of our knowledge, this is the first study focusing on the effect of ACEI/ARB on the severity of the AMI in diabetic hypertensive patients.

The current study found that 52.0% (1300/2501) of patients with diabetic hypertension and diagnosed with OCAD for the first time did not use ACEI/ARB therapy. Notably, the proportion of patients treated with ACEI/ARB during hospitalization and long-term follow up in the ACEI/ARB(-) group was 51.0% and 41.3%, respectively, which were substantially lower than the proportions in the ACEI/ARB(+) group (83.9% and 67.0%, respectively). A study from the United States (from six states, 57,1483 participants) has shown that about 52.5% of patients with diabetic hypertension were non-adherent to ACEI/ARB therapy, which was related to an increased risk for diabetes-related rehospitalizations [19]. All these findings suggest that the real-world use of ACEI/ARB is seriously insufficient worldwide and the underutilization of ACEI/ARB may lead to poor clinical outcomes. We analyzed the factors associated with the use of ACEI/ARB and found that patients who have previously used CCB were less likely to receive ACEI/ARB therapy before the hospital admission, suggesting that CCB might affect ACEI/ARB’ first-line status in diabetic hypertensive patients. Based on the recommendations of the current guidelines [14–16], we believe the first-line treatment status of ACEI/ARB in patients with diabetic hypertension still need to be emphasized.

## Limitations

First, this is a single-center study although including a large sample size; thus, generalization of the findings should be cautious. Second, this is a retrospective observational study. The information on the dosage and duration of ACEI/ARB was limited. Prospective cohort studies are required to confirm our findings.

## Conclusions

Use of ACEI/ARB therapy for diabetic hypertensive patients before the initial diagnosis of OCAD was significantly associated with lower incidence of AMI, improved heart function, smaller infarct size, and lower incidences of non-fatal stroke and composite MACCE. No prior ACEI/ARB therapy was significantly and independently associated with non-fatal stroke and composite MACCE. ACEI/ARB therapy was largely underutilized in diabetic hypertensive patients.

## Data Availability

The datasets used and/or analyzed during the current study are available from the corresponding author on reasonable request.

## Abbreviations

ACEI/ARB: Angiotensin-converting-enzyme inhibitors/angiotensin receptor blockers
OCAD: Obstructive coronary artery disease
MACCE: Major adverse cardiac and cerebral event
AMI: Acute myocardial infarction
Myo: Myoglobin
CKMB: Creatine kinase MB
cTnI: Cardiac troponin I
RAAS: Renin–angiotensin–aldosterone system
BMI: Body mass index
FBG: Fasting blood glucose
RBG: Random blood glucose
HGB: Hemoglobin
eGFR: Estimated glomerular filtration rate
LDL-C: Low-density lipoprotein cholesterol
hsCRP: High sensitivity C reactive protein
LVESD: Left ventricular end-systolic diameter
LVESV: Left ventricular end-systolic volume
LVEF: Left ventricular ejection fraction
LVFS: Left ventricular fraction shortening
SV: Stroke volume
CCB: Calcium channel blocker
T_2_DM: Type 2 diabetes mellitus
